# Acute Compartment Syndrome in Orthopedics: Causes, Diagnosis, and Management

**DOI:** 10.1155/2015/543412

**Published:** 2015-01-19

**Authors:** Hasnain Raza, Anant Mahapatra

**Affiliations:** Our Lady of Lourdes Hospital, Drogheda, Ireland

## Abstract

Almost all orthopaedic surgeons come across acute compartment syndrome (ACS) in their clinical practice. Diagnosis of ACS mostly relies on clinical findings. If the diagnosis is missed and left untreated, it can lead to serious consequences which can endanger limb and life of the patient and also risk the clinician to face lawsuits. This review article highlights the characteristic features of ACS which will help an orthopaedic surgeon to understand the pathophysiology, natural history, high risk patients, diagnosis, and surgical management of the condition.

## 1. Introduction

Almost all orthopaedic surgeons come across acute compartment syndrome (ACS) in their clinical practice. Dr. Volkmann, a German doctor in 1881, described ACS by reporting the hand contracture which was a consequence of this particular condition [1]. In 1888, Petersen for the first time reported the management of ACS [2]. The compartment syndrome is mostly diagnosed on variation in clinical symptoms and signs in sequential examinations. If the diagnosis is missed and left untreated, it can lead to serious damage to the soft tissues of the limb including muscles, nerves, and vessels. It can sometimes result in limb loss or even Loss of Life. An orthopaedic surgeon must have an understanding of this condition, including specific injuries and specific group of patients which are more vulnerable in getting ACS. A surgeon should understand the basics of compartment syndrome including pathophysiology, epidemiology, diagnosis, and management [3].

## 2. Pathophysiology

Compartment syndrome is defined as a condition in which a closed compartment's pressure increases to such an extent that the microcirculation of the tissues in that compartment is diminished [4].

Two factors are responsible for this condition, either a decrease in a compartment volume or an increase in the contents of a compartment, or sometimes both of these factors act at the same time. ACS develops when the intracompartmental pressure (ICP) exceeds the venous capillary pressure. Elevated ICP results in raised pressure at the venous capillary end and increases hydrostatic pressure, leading to arteriolar compression [5]. The microcirculation compromised due to arteriolar compression, hence reducing or diminishing perfusion of the tissues. Inadequate perfusion and oxygenation result in soft tissue ischemia and anoxia and death of the cells. The most ischemic vulnerable tissue in a compartment is skeletal muscle [6]. Extent of muscle death is dependent on the duration of ischemia, temperature of the tissues, and the available residual microcirculation. Sufficient collateral blood supply and lower local temperature slow down the ischemic process [7]. Rorabeck and Clarke showed that the duration of increased pressure is significant in the return of neurological function. Pressures 40 to 80 mm Hg sustained for 4 hours do not cause permanent nerve dysfunction, but, when applied for 12 hours or more, permanent neurological changes occurred [8]. In conclusion, the amount of skeletal muscle necrosis is directly proportional to duration of ischaemia and inversely proportional to temperature.

## 3. Epidemiology

Acute compartment syndrome usually occurs in traumatized patients who have such injuries which distract the clinician from diagnosing ACS. In management of these patients, the clinician should have a high degree of suspicion. The most common site of ACS is leg which is followed by forearm, arm, thigh, foot, gluteal region, hand, and abdomen.

Various risk factors are related to compartment syndrome and age is one of the important factors. Younger patients are more prone to get ACS as compared to elderly patients with the same nature of trauma [9]. Another risk factor is the type and site of injury. Closed tibial shaft fracture is the most common cause of compartment syndrome and is comprised of one-third of all cases of ACS. One-fourth of the cases result from blunt and crushed soft tissue limb trauma while radius ulna shaft fractures are responsible for 20 percent of the cases. Foot injuries in road traffic accidents account for 6% of all cases of ACS [10], while the incidence is even lesser in lower leg injuries [11]. Revascularization after acute arterial injury or obstruction can also result in ACS; hence in most of cases patients need fasciotomy after revascularization [12].

Males are more prone to develop ACS which is ten times higher than females. Incidence of ACS in open and closed fractures is equal. Other less common causes of traumatic ACS include burns and blunt or crushing trauma to the limb. ACS can develop by poor positioning of legs in prolonged surgical procedures, particularly lithotomy position [13]. Excessive exercise by athletes or nonroutine physical activity or overuse in nonathletes can also lead to acute compartment syndrome (ACS) of the leg which needs urgent medical attention [14]. Acute compartment syndrome can also result from nonaccidental causes like medical conditions which include nephrotic syndrome, viral myositis, hypothyroidism, bleeding disorders, malignancies, and diabetes mellitus [15]. Diabetes-associated muscle infarction (DMI) is a condition in diabetics which results from compartment syndrome [16]. Ruptured Baker's cyst is also reported as a rarer cause of ACS [17].

## 4. Clinical Diagnosis

Compartment syndrome is mostly diagnosed clinically. Lack of knowledge and inadequate practical exposure lead to delayed or missed diagnosis. Examination should be done serially more at various times than at any one specific point of time for making any definitive diagnosis. It is preferred to have one surgeon who should perform serial assessment and make the diagnosis. If the sign and symptoms are equivocal, then it is preferred to take a second opinion from the senior colleague. One of the most important prognostic factors for outcome is the time of development of ACS to the time of diagnosis and the time of surgical treatment.

The five “P's” mentioned in the literature for compartment syndrome are pain, paralysis, paresthesia, pallor, and pulselessness [18]. Though all of the mentioned clinical signs and symptoms are important clinical findings, mostly all are not present in every case, and in fact presence of pulselessness indicates that it is already too late to get good outcome. The cardinal symptom of ACS in an awake patient is “pain out of proportion.” Pain at rest and with passive stretch is almost always found in evolving ACS. But if the ACS is already established and ends up in late stage, pain may not be the clinical finding as the pain receptors and nerve fibers face ischemic necrosis and death. Moreover, pain can be absent in regional anaesthetised patients and sedated and relaxed patients in ICU.

The first sign of nerve ischemia is paraesthesia which is followed by hypoaesthesia, anaesthesia, paresis, and paralysis. Sensory assessment should be done by pinprick testing, light touch, and two-point discrimination in awakened patients. Motor deficit in the affected limb can be due to ischemia of nerves and/or muscles or secondary to pain. Complete paralysis is found in late stage of compartment syndrome and indicates irreversible damage to nerves and/or muscles.

Pulselessness in ACS is also a late finding. In ACS, pressure in the compartments is not usually high enough to compress arteries. Loss of pulse and presence of Pallor limb could be an indication of direct arterial injury. Capillary refill is mostly present even in well-developed ACS if there is no direct arterial injury.

The only clinical sign in impending ACS could be massive swelling of the limb with firm compartments. In unconscious patients most of the clinical findings cannot be elicited; hence it is necessary to check compartment pressure by devices.

## 5. Intracompartment Pressure Monitoring

**Figure 1:**
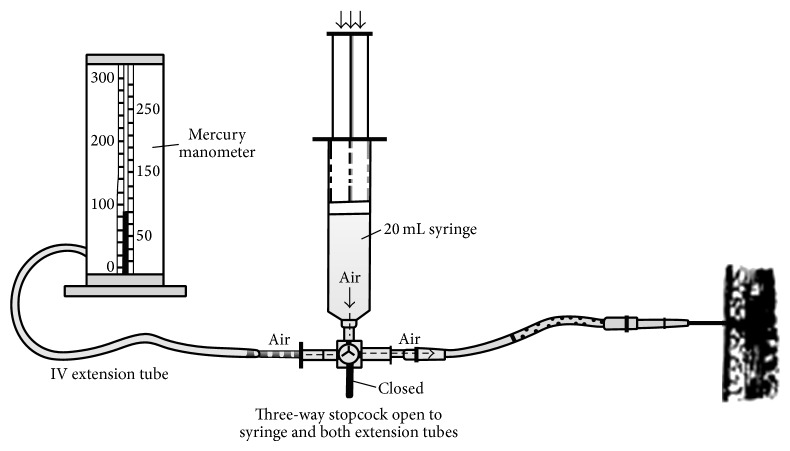
Manual setup for intracompartmental pressure measurement (Campbell Operative Orthopaedics, 11th Edition).

**Figure 2:**
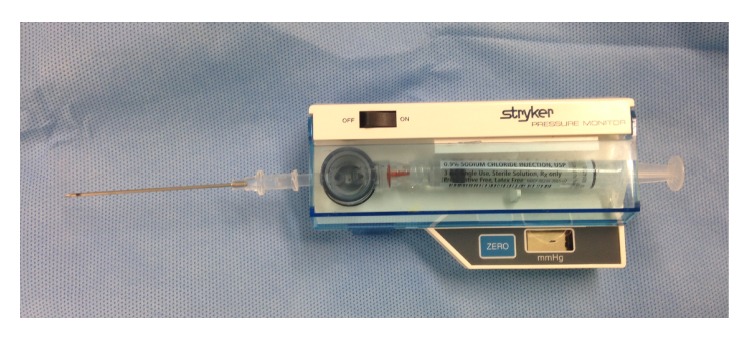
Stryker digital device.

**Figure 3:**
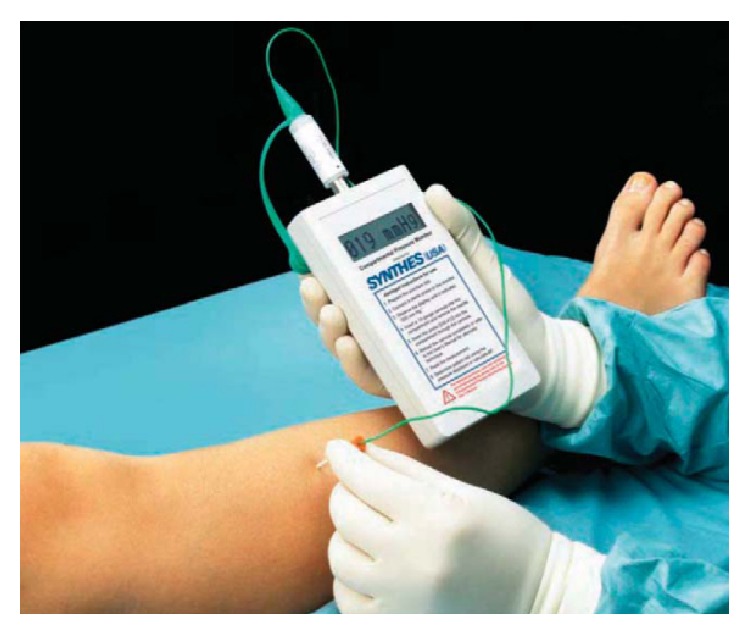
Synthes (West Chester, PA) hand-held compartment pressure monitor.

Various techniques and devices for intracompartment pressure measurement (ICP) are mentioned in the literature. The measurement of ICP for diagnosis of ACS in an awakened patient is controversial. ICP is nearly 8 mm Hg in resting adults and almost double in paediatrics [19]. Various techniques for measuring ICP include hand-held monitor for single pressure readings, Stryker needle with side portal, and regular needle with arterial line setup. If more sophisticated equipment is unavailable, compartment pressure can be measured using intravenous tubing, a three-way stopcock, a syringe, and a mercury manometer, as described by Whitesides et al. [20] (Figure 1). Boody and Wongworawat compared three commonly used devices for measuring compartment pressures which included Stryker Intracompartmental Pressure Monitor System (Figure 2), arterial line manometer, and Whitesides apparatus (Figure 3). Boody et al. reported that arterial line manometer was the most accurate manometer which was followed by Stryker device and use of side port needles gave better results than the straight needles.

Compartment pressures were found different at various locations within compartments in relation to injury site; hence there is a relationship between ICP and distance from the fracture site. Heckman et al. suggested that pressure should be measured in different sites in all compartments but within 5 cm of the fracture site [21].

Various authors mentioned different values of compartment pressure considered to be the threshold for surgical decompressive fasciotomy. Matsen et al. used an absolute value of 45 mm Hg for diagnosis of ACS and indication for fasciotomy while 30 mm Hg was used by Mubarak et al. [22, 23]. McQueen and Court-Brown suggested that if the difference between diastolic blood pressure and ICP was less than 30 mm Hg, it was highly suspicious of ACS and needed to be decompressed [24]. Gelberman et al. also recommended compartment fasciotomies for compartment pressures greater than 30 mm Hg [25]. Other authors have recommended fasciotomy for pressures greater than 40 mm Hg or delta values of 40 mm Hg (the difference between mean arterial pressure and the compartment pressure) [26]. Establishing diagnosis on these measurements alone in a conscious patient may lead to unnecessary surgery [27]. McQueen et al. reported in a retrospective study 93% sensitivity of ICP monitoring in suspected ACS with an estimated specificity of 98%, an estimated positive predictive value of 93%, and an estimated negative predictive value of 99% [28]. While Whitney et al. mentioned 35% false-positive rate for the diagnosis of ACS in patients with tibial shaft fractures on one time ICP measurement under anaesthesia prior to fixation of tibial fractures, in fact clinically they did not have clinical evidence of compartment syndrome pre- and postoperatively and fasciotomy was not performed [29]. Literature supports continuous ICP monitoring rather than one time measurement for establishing diagnosis of ACS.

Patients who are not awake and alert or who have been given regional block for anaesthesia or postoperative analgesia must be observed more carefully as clinical signs and symptoms cannot be picked up [30]. The clinician should have the high index of suspicion for ACS diagnosis in such patients and should not make delay in monitoring and measuring ICP with the available devices.

## 6. Infrared Spectroscopy

A new technique which is called near infrared spectroscopy (NIRS) is a noninvasive and continuous technique. It is based on absorption of light in near infrared spectrum which corresponds to oxygenated and deoxygenated hemoglobin. Assessment of tissue oxygenation was done by comparing with the oxyhaemoglobin and deoxyhaemoglobin concentration in venous blood. Garr et al. demonstrated an inverse relation between compartment pressure and oxygenation in an animal model [32].

## 7. Intracompartment pH Monitoring

In addition to clinical features and ICP measurement Elliot described the role of intramuscular (IM) pH monitoring in the diagnosis of ACS. He reported the higher specificity in measuring IM pH which was found to be 80% with pH less than 6.38 while the specificity in monitoring ICP was found to be 27% to 30%. He recommended that the patients with ACS can be early and accurately identified using IM pH monitoring and subsequently reduce the morbidity associated with ACS [33].

## 8. Fasciotomy

Once the diagnosis of ACS is established, then the surgical decompressive fasciotomy should be performed urgently but a good surgical technique is mandatory. Once the decision for fasciotomy is made, the theatre arrangements should be expedited. In the meanwhile, keep leg elevated in order to increase venous return and decrease swelling. All dressings should be loosened or removed if possible. Send blood samples for baseline investigations and group and screen for possible transfusion in postoperative period.

There are various techniques of fasciotomy of leg in the literature, which include single incision fasciotomy with fibulectomy, single incision fasciotomy without fibulectomy, and the most common surgical approach two-incision fasciotomy with anterolateral and posteromedial incisions.

**Figure 4:**
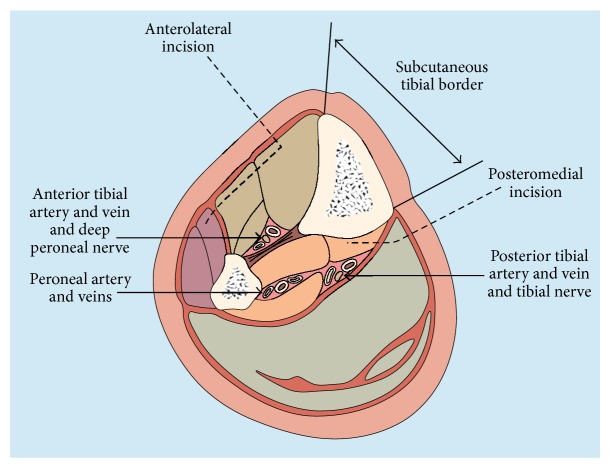
Cross section through leg showing site of fasciotomy incisions to decompress all four compartments [31].

In two-incision technique, the anterolateral incision is made to approach the anterior and lateral compartments. It is midway between the tibial crest and the fibular head (Figure 4). Incision starts 5 cm distal to fibular head and extends up to 5 cm proximal to lateral malleolus. Fascia of the anterior and lateral compartments should be released through this incision. Surgeons should be careful about superficial peroneal nerve which comes across around 10–12 cm proximal to the lateral malleolus while exiting from the fascia. This approach could expose periosteum of the lateral malleolus and the peroneal tendons. The viability of the muscles should be assessed after fasciotomy. The pink/red colour of the muscle and presence of contraction on stimulus are an indication of viable muscle. All nonviable muscles should be excised. The exposed tendons, periosteum, and the muscles should be kept moist to avoid desiccation of the tissues and prevent infection [31]. The second incision is posteromedial incision which is made 2 cm posterior to the medial border of the tibia. This incision is utilised to release the superficial and deep posterior compartments and approach the muscles in these compartments for assessment of viability. Soleus insertion should be released to adequately decompress the posterior compartment. Surgeons should try to avoid sacrificing the saphenous nerve and vein while doing the procedure.

**Figure 5:**
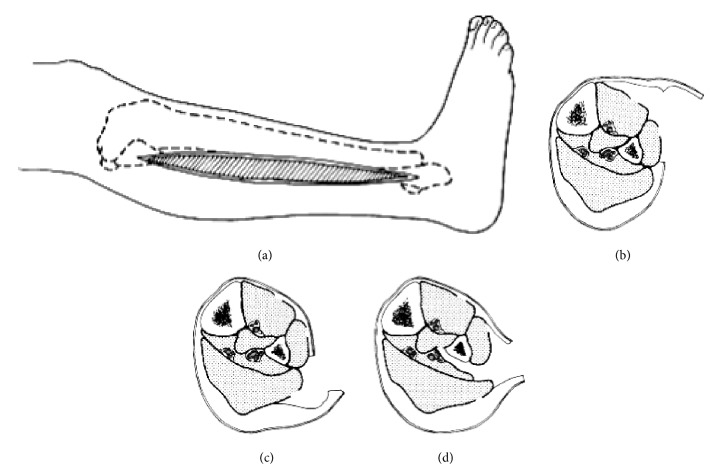
One-incision technique without fibulectomy. (a) Lateral skin incision from fibular neck 3 to 4 cm proximal to lateral malleolus. (b) Skin is undermined anteriorly, and fasciotomy of anterior and lateral compartments is performed. (c) Skin is undermined posteriorly, and fasciotomy of superficial posterior compartment is performed. (d) Interval between superficial posterior and lateral compartments is developed. Flexor hallucis longus muscle is dissected subperiosteally off fibula and retracted posteromedially. Fascial attachment of posterior tibial muscle to fibula is incised to decompress muscle (redrawn from [34]).

The single incision technique is successful in experienced hands but it is less popular (Figure 5). Maheshwari et al. reported excellent outcome in their case series of 58 legs which had single incision fasciotomy. A longitudinal incision is made over the fibula extending 5 cm distal to fibular head and 5 cm proximal to lateral malleolus. Through this approach, the anterior, lateral and superficial posterior compartments are released first and then followed by release of the deep posterior compartment at the posterolateral fibular insertion site of lateral intermuscular septum. This approach risks the peroneal nerves and vessels when entering into deep posterior compartment. The surgeon must incise the lateral intermuscular septum at its fibular insertion.

Though fibulectomy through a single lateral incision was considered a popular technique for four-compartment fasciotomy of the leg, now it is replaced by two-incision fasciotomies due to less morbidity [35].

**Figure 6:**
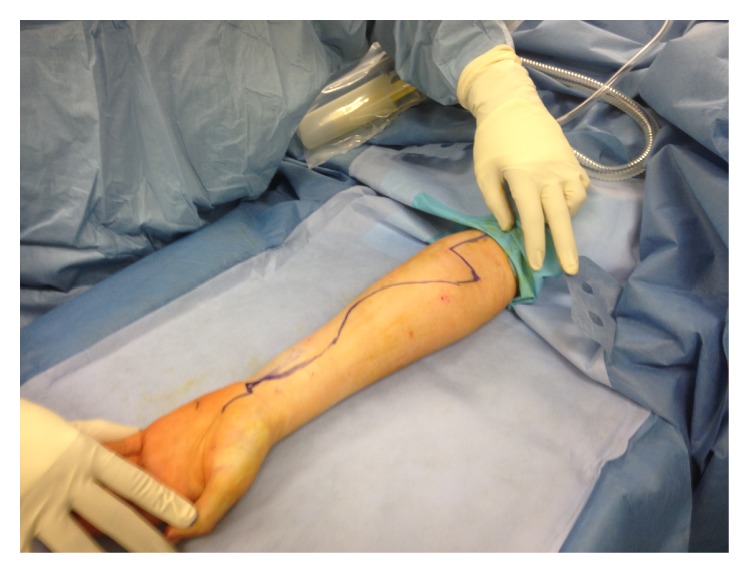
The volar S shaped incision including proximal palm to decompress carpal tunnel.

The second common site for developing compartment syndrome is forearm. There are four compartments in forearm: volar, dorsal, Mobile wad of Henry, and the pronator quadratus [36]. The forearm compartments are not completely independent of one another as in the leg. Hence individual compartments do not need to be individually definitely addressed. The volar compartment is most commonly involved and needs decompression. Various incision patterns have been described in the literature, including lazy S shaped and curved incisions. The incision should be ulnar aspect at the wrist to avoid keeping exposed radial artery and median nerve which are superficial at the wrist. The volar incision should always include proximal palm to release the transverse carpal ligament of carpal tunnel (Figure 6).

After releasing the flexor digitorum superficialis, the deep volar musculature such as flexor digitorum profundus, pronator quadratus, and flexor carpi ulnaris should also be decompressed. Following volar compartment release, the dorsal and mobile wad compartments' pressures should be measured. Mostly the volar compartment decompression releases the pressure from the extensor compartment as well. For releasing the dorsal compartment, a longitudinal incision was made which extends from 4 cm distal to lateral epicondyle to Lister tubercle [37].

**Figure 7:**
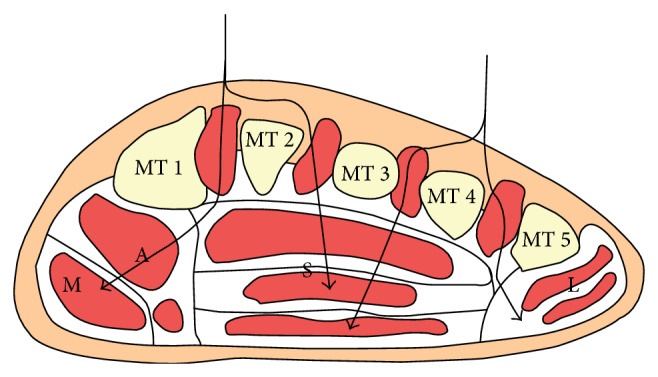
Anatomical section view of the forefoot. The dorsal approach uses one or two longitudinal incisions. It facilitates access to the interosseus and adductor compartments. MT = metatarsal; M = medial compartment; A = adductor compartment; S = superficial compartment; L = lateral compartment.

For isolated calcaneal compartment syndrome in which the planter nerves and vessels are compressed, a single planter incision should be made from medial side of heel and foot. This approach starts with an incision on planter side of the first metatarsal. The abductor hallucis which is a muscle in medial compartment should be longitudinally split. Wounds can be closed by delay primary closure or get healed by secondary intention. Mubarak and Owen described a dorsal approach for interosseus compartments release which are the most affected compartments in ACS of foot. This approach consists of two dorsal incisions over the second and fourth metatarsals, keeping maximum possible width of skin bridge to avoid skin flap necrosis [38] (Figure 7). This dorsal approach helps in accessing all compartments and provides adequate exposure for fracture fixations. The surgeon should be careful about superficial veins and nerves.

Fasciotomies are not benign procedures as they impair long term calf muscle pump function in patients with and without vascular injuries. These patients can develop chronic venous insufficiency following trauma and fasciotomy [39].

## 9. Wound Management after Treatment

Though fasciotomy is a limb saving procedure it can carry significant morbidity. The fasciotomy incisions can lead to large, unsightly, and chronic wounds. At 48 to 72 hours after fasciotomy, the patient should be taken back to theatre for relook and debridement of nonviable tissues. If there are no residual necrotic tissues, the skin is loosely closed. If complete closure is not possible, then assisted closure methods should be applied.

A popular method of assisted closure of fasciotomy wounds is negative pressure wound therapy (NPWT) [40]. NPWT dressings are a closed system whereby a vacuum applies subatmospheric pressure to a wound through a porous foam dressing, reducing extravascular pressure and oedema within the compartment, leading to improved circulation, granulation, and approximation of wound edges, as well as less bacterial colonisation [41]. NPWT reduces the risk of infection but it ends up with high chance of skin grafting [42].

**Figure 8:**
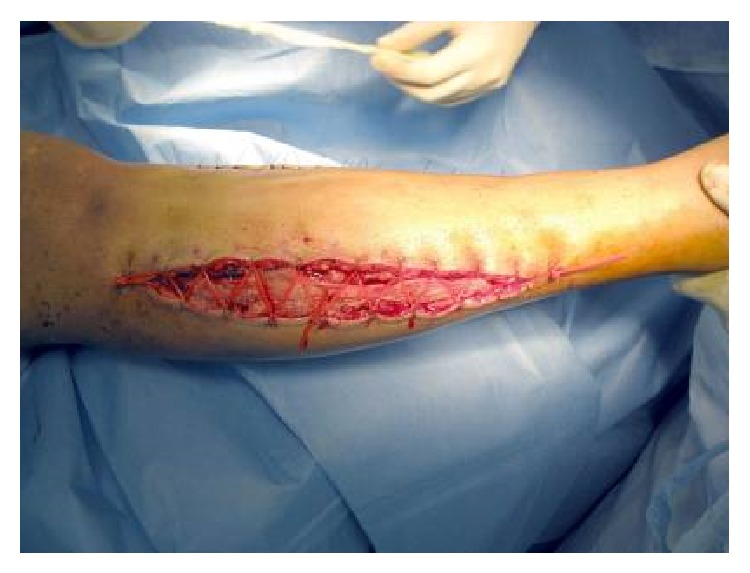
Dynamic wound closure using the vessel loop or shoelace technique.

Dynamic wound closure using the vascular loop or shoelace technique has also been described as a viable management option (Figure 8). This method entails approximation of wound edges using vascular loops anchored by skin staples and gradually tensioning them across the wound margins [43]. This method helps in avoiding skin grafting.

## 10. Medicolegal Aspect

There is significant medicolegal aspect of ACS and its outcome in clinical practice. Bhattacharyya and Vrahas reviewed all cases and claims related to ACS filed with a large insurer over a 23-year period. The data showed that greater than 50% decided against doctors [44]. Shadgan et al. reported fifty-five percent (35/64) of legally completed cases which were ruled in favour of the patients [45].

Reverte et al. mentioned significantly high incident of delayed union or nonunion of tibial shaft fractures with compartment syndromes. They reported 55% nonunion or delayed union in ACS versus 17.8% in fractures without ACS in a meta-analysis study. It is highly recommended to inform patients about increased chance of fracture healing complications [46].

## 11. Conclusion

ACS is one of the few orthopaedic emergencies which can lead to limb and life threatening outcome if there is delay in diagnosis and treatment. All physicians involved in dealing with such emergencies should be hypervigilant and there should be a low threshold for fasciotomy.
